# Microbial succession in human tissues postmortem: insights from 2bRAD-M sequencing

**DOI:** 10.1128/spectrum.02666-24

**Published:** 2025-11-17

**Authors:** Xin Huang, Jianye Zeng, Fan Yang, Yanan Liu, Ji Chen, Hongyan Wang, Shilin Li, Chengtao Li, Suhua Zhang

**Affiliations:** 1 Institute of Forensic Science, Fudan University 12478, Shanghai, People's Republic of China; 2 School of Forensic Medicine and Science, Fudan University 12478, Shanghai, People's Republic of China; 3 Institute of Forensic Science, Shanghai Municipal Public Security Bureau/Key Laboratory of Crime Scene Evidence 359357 , Shanghai, People's Republic of China; 4 The State Key Laboratory of Genetic Engineering and MOE Key Laboratory of Contemporary Anthropology, School of Life Sciences, Fudan University 12478, Shanghai, People's Republic of China; American Type Culture Collection, Manassas, Virginia, USA

**Keywords:** thanatomicrobiome, microbial succession, postmortem interval, 2bRAD-M sequencing

## Abstract

Microbial communities play a crucial role in decomposition, yet their patterns in human tissues remain underexplored. Most previous research has often focused on animal models such as mice and swine, with limited studies on human samples, primarily targeting specific environments like the gut and skin. Consequently, gaps persist in understanding postmortem microbial dynamics within internal human organs. The 2bRAD-M sequencing technology offers a powerful approach for human thanatomicrobiome research, overcoming key limitations of 16S rRNA and metagenomic sequencing methods. In this study, we used 2bRAD-M to profile microbial succession across seven human tissues—heart, liver, spleen, lung, kidney, calf muscle, and gut—at various postmortem intervals (PMIs). Significant variations in microbial community composition were observed across organs and decomposition stages, with *Proteobacteria* dominating early and *Firmicutes* later. A comparison of frozen and unfrozen cadavers (PMI 1–7 days) revealed divergent microbial shifts in the liver and spleen, while other tissues exhibited limited variation. These findings highlight complex, organ-specific microbial trajectories and suggest that microbial signatures could serve as biomarkers for PMI estimation. This research deepens our understanding of the microbial succession within internal human organs postmortem and contributes to elucidating the identity and role of microorganisms in human decomposition.

**IMPORTANCE:**

Humans host a diverse array of microbial communities that play a crucial role in the decomposition process after death. Understanding these postmortem microbial dynamics is essential, as they offer valuable insights into the progression of decomposition with significant implications for forensic science. The role of microorganisms in corpse decomposition has gained increasing attention in both forensic and ecological research, but studies in this area remain in their early stages, requiring further in-depth exploration. This work pioneers the use of 2bRAD-M sequencing to investigate microbial changes across various human organs over increasing postmortem intervals. By enhancing knowledge of postmortem microbiota dynamics, the study contributes to refining and improving the accuracy of forensic methodologies.

## INTRODUCTION

The human body hosts an extensive diversity of microorganisms that inhabit every ecological niche. The Human Microbiome Project has emphasized the intricate relationship between human health and microbial components (1), documenting their distribution and function throughout life. Despite these advances, a critical gap remains in understanding the alterations that occur in microbial communities after death. Following the host’s demise, microorganisms persist, and death itself acts as a major ecological disturbance to the microbiota (2). The postmortem microbial community, or thanatomicrobiome, is characterized by a successional process in which trillions of microbes colonize, reproduce, and eventually perish both internally and externally, leading to temporal shifts in community composition as decomposition progresses (3).

Following death, the onset of hypoxia triggers the release of intracellular components, initiating autolysis. This process liberates nutrient-rich cellular materials that are rapidly exploited by microorganisms, facilitating their proliferation and driving the decomposition cascade (4, 5). Cadaveric decomposition process can be broadly categorized into five main stages: fresh decay, bloat, active decay, advanced decay, and skeletonization (6). Previous studies have demonstrated that microbial community composition varies across anatomical areas and changes over time (7). During the initial days to weeks of decomposition, putrefaction is primarily driven by bacteria, with the microbial communities rapidly transitioning from aerobic to anaerobic taxa (8). Research has reported a decrease in microbial richness, diversity, and evenness over time (9). For instance, Hauther et al. (10) investigated the postmortem succession patterns of colonic bacterial communities in six cadavers, revealing an exponential decline in the relative abundance of *Bacteroidetes* and *Lactobacillus*. This decline might be attributed to gas accumulation released during autolysis, leading to oxidative stress in anaerobic bacteria (11). These findings highlight the potential of the postmortem microbiome analysis in forensic science, particularly for the estimation of the postmortem intervals (PMIs) (12–15).

Although promising, research on human postmortem microbial succession remains limited due to the scarcity of cadaveric samples. Consequently, many studies have relied on model animals such as swine and juvenile rodents (16–18). Human research has mainly focused on the microbiomes of the gastrointestinal tract or skin (19), leaving microbial succession in other tissues and organs underexplored. Notable exceptions include Can et al., who profiled thanatomicrobiome in internal organ tissues from 11 cadavers, finding the obligate anaerobe *Clostridium* in cadavers at varying postmortem intervals and observing greater *Lactobacillus* abundance in cadavers at shorter PMIs (5). Similarly, Javan et al. sequenced samples from the brain, heart, liver, spleen, blood, and oral cavity of 27 cadavers, revealing gender-specific microbial differences and identifying *Firmicutes* as a potential biomarker across thanatomicrobiome communities (20). These findings highlight the urgent need for comprehensive characterization of postmortem microbial succession in diverse internal body sites.

Currently, 16S rRNA V3-V4 region sequencing and metagenomic sequencing represent the dominant approaches for microbiome analysis. However, 16S rRNA sequencing often suffers from insufficient taxonomic resolution, and metagenomic sequencing frequently encounters high levels of host DNA contamination (21, 22). To overcome these challenges, the recently developed 2bRAD-M technology offers improved accuracy and sensitivity in microbial detection (23). Our previous work has demonstrated the suitability of this technique for human thanatomicrobiome research (24).

In this study, we used 2bRAD-M sequencing to examine the microbiota in seven distinct body sites (including hearts, livers, spleens, lungs, kidneys, calf muscles, and guts) from 32 cadavers at different PMIs. By characterizing microbial succession across different organs, we aim to expand empirical knowledge of thanatomicrobiome dynamics. Furthermore, identifying organ-specific microbial signatures may provide valuable data and insights for improving forensic methods, particularly in the estimation of PMIs.

## MATERIALS AND METHODS

### Sample collection

**Fig 1:**
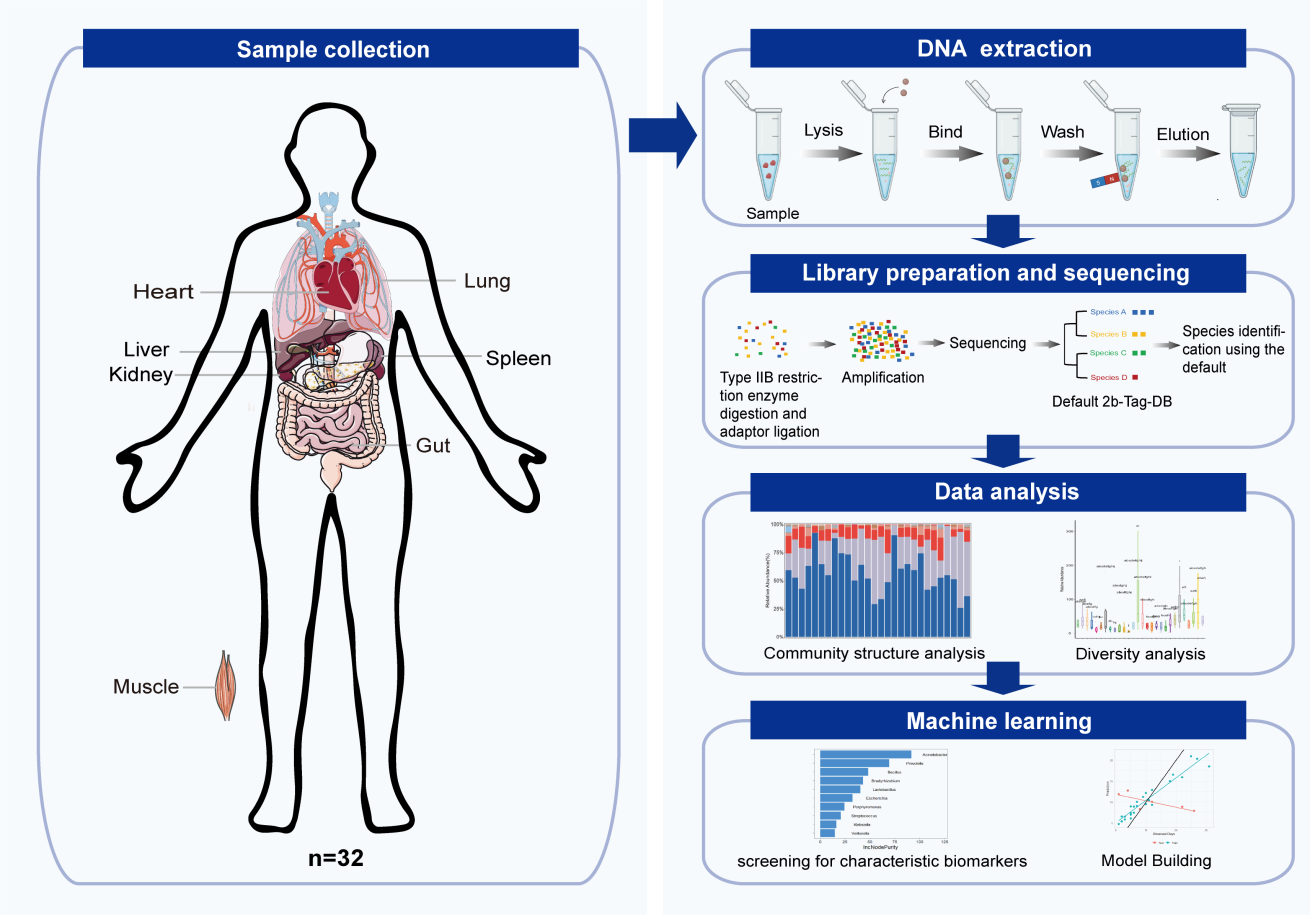
A schematic overview of the study workflow.

A schematic overview of the study workflow is shown in Fig. 1. Tissue samples were collected from the hearts, livers, spleens, lungs, kidneys, calf muscles, and guts of 32 cadavers, comprising 26 males and 6 females, aged 23 to 87 years (Table 1). These cadavers were transported directly from the crime scenes to the morgue or laboratory without undergoing burial or other intermediary processes, ensuring that they represent real case samples. The cadavers were selected based on specific criteria, excluding those with prior microbial infections or antibiotic treatment. PMI for these samples ranged from 1 to 31 days. Of the 32 cadavers, 11 were processed as fresh samples and 21 as frozen samples. The frozen cadavers were placed in a refrigerated cabinet set to −15 to −20°C within 2 h after death anfrozend remained at that condition until tissue collection. Detailed demographic and clinical data, which were used to correlate microbial variations, are provided in Table S1. To minimize cross-contamination, the samples were meticulously dissected using sterile scalpels, with all instruments sterilized between each dissection. Sampling was conducted in a controlled environment with consistent temperature and humidity, maintaining sterile conditions throughout the procedure to preserve sample integrity.

**TABLE 1:** Detailed summary of the human cadavers included in the study

Characteristic	
Sex	Number of individuals
Male	26
Female	6
Postmortem intervals (days)	Number of individuals
1–7	16
9–12	7
15–31	9
Storage conditions	Number of individuals
Room temperature	11
Frozen	21
Cause of death	Number of individuals
Coronary heart disease	7
Traffic injury	6
Craniocerebral injury	4
Sudden death	3
Traumatic shock	2
Drug intoxication	1
Drowning	1
Electric shock	1
Hanging	1
Septic shock and multiple organ failure	1
Aortoclasia	1
Acute pulmonary embolism	1
Injury by fall from height	1
Stomach tumor	1
Unknown	1
Age	Years
Median	52.5
Range	23–78
Organ	Number of individuals
Heart	32
Liver	32
Spleen	32
Lung	32
Kidney	32
Gut	32
Calf muscle	32

### DNA extraction, library preparation, and sequencing

Genomic DNA was extracted from the collected samples using the CretMag Multi-Sample DNA Kit, following the manufacturer’s protocol. Approximately 20 mg of tissue was used for each sample to ensure consistency across extractions. For library preparation, the DNA samples were digested with BcgI enzyme at a concentration of 2 U/µL for 3 h at 37°C. The BcgI enzyme was selected for its ability to generate specific fragments suitable for 2bRAD-M sequencing. The efficiency of the digestion process was confirmed by 1% agarose gel electrophoresis. Following digestion, the DNA fragments were ligated using T4 DNA ligase (400 U/µL) in a ligation reaction system maintained at 4°C for 12 h. The ligation products were then subjected to PCR amplification with an initial denaturation at 95°C for 3 minutes, followed by 28 cycles of denaturation at 98°C for 5 seconds, annealing at 60°C for 20 seconds, and extension at 72°C for 10 seconds. Final products were purified using the QIAquick PCR Purification Kit (Qiagen, CA) and sequenced on the Illumina NovaSeq PE150 platform to ensure high-throughput data acquisition. Sequence quality was assessed using FastQC to ensure high-quality reads for subsequent analysis. 2bRAD-M was carried out at Qingdao OE Biotech Co., Ltd. (Qingdao, China).

### Data processing and analysis

The initial raw reads were filtered using Cutadapt v.3.5 (25) software to isolate digested fragments based on BcgI restriction enzyme recognition sites, referred to as “enzyme reads.” Clean reads were then obtained using Trimmomatic, discarding sequences with an N-base ratio exceeding 8% or a Phred score below 20. The resulting clean reads were mapped to the 2bRAD-M database using Bowtie2 v.2.2.9 (26) for microbial annotation. For further refinement, a secondary library construction was performed using potential microbial genomes, and the updated database was re-queried with BLAST+ to obtain clean reads for each sample. Diversity analyses were conducted using QIIME2 v.2020.11 (27), with alpha-diversity assessed through Chao1 (28), Shannon (29), and Simpson indices. The Chao1 index estimates species richness, providing insight into the number of distinct species present, while the Shannon and Simpson indices measure species abundance and evenness, reflecting community complexity and dominance dynamics. These metrics were selected for their ability to capture nuances in community structure, offering a comprehensive view of microbial succession after death. Beta-diversity was used to compare the microbial composition similarities in each group. The unweighted UniFrac distance matrix was used for principal coordinates analysis (PCoA). Statistical significance was set at *P* < 0.05.

### Random forest regression modeling

Processed feature tables were constructed from 2bRAD-M sequencing data, incorporating variables such as relative abundance, taxonomic levels, and sample metadata. These tables served as input for random forest regression modeling conducted using RandomForest v.4.7.1.1 in R v.4.1.3. To ensure robust evaluation, the data were stratified so that models were trained and tested independently on different tissues. For each tissue group, samples were randomly divided into a training set (75%) and a test set (25%). Random forest regression was employed to model the relative abundance of microbiota at various taxonomic levels in relation to PMI. The relative importance of each bacterial species to the model’s predictive accuracy was evaluated using the Mean Decrease Gini index. A fivefold cross-validation technique was applied using the caret v.6.0.94 to examine the relationship between the number of bacterial species included in the model and the prediction error. Bacterial species were selected based on their importance ranking to construct the final PMI inference model. The model’s performance was assessed using the mean absolute error (MAE) and goodness of fit (*R*^2^). Data visualization was performed with ggplot2 v.3.5.1.

## RESULTS

### Overall characterization of sequencing data

In this study, a total of 219 tissue samples were collected and analyzed from 32 human cadavers. Based on PMI, samples were divided into three groups: group 1 (PMI 1–7 days), group 2 (PMI 9–12 days), and group 3 (PMI 15–31 days). To account for the presence of both fresh and frozen conditions in group 1, this group was further subdivided into group 1A (fresh) and group 1B (frozen). In the accompanying figure, each sample is annotated by tissue type and group, such as “Heart1A,” representing heart tissue from group 1A.

From 2bRAD-M sequencing, we obtained 4.53–10.06 million raw reads per sample. After removing low-quality reads, clean reads accounted for 73.2%–93.9% of raw data. Negative controls from extraction and library preparation (NC-EX-16, NC-EX-20, NC-EX-28, NC-JK-16, NC-JK-20, NC-JK-28) yielded only 8,360–313,355 raw reads (Table S2), which is substantially lower than the positive samples’ yield.

### Dynamics of the microbial community with increasing postmortem interval

**Fig 2:**
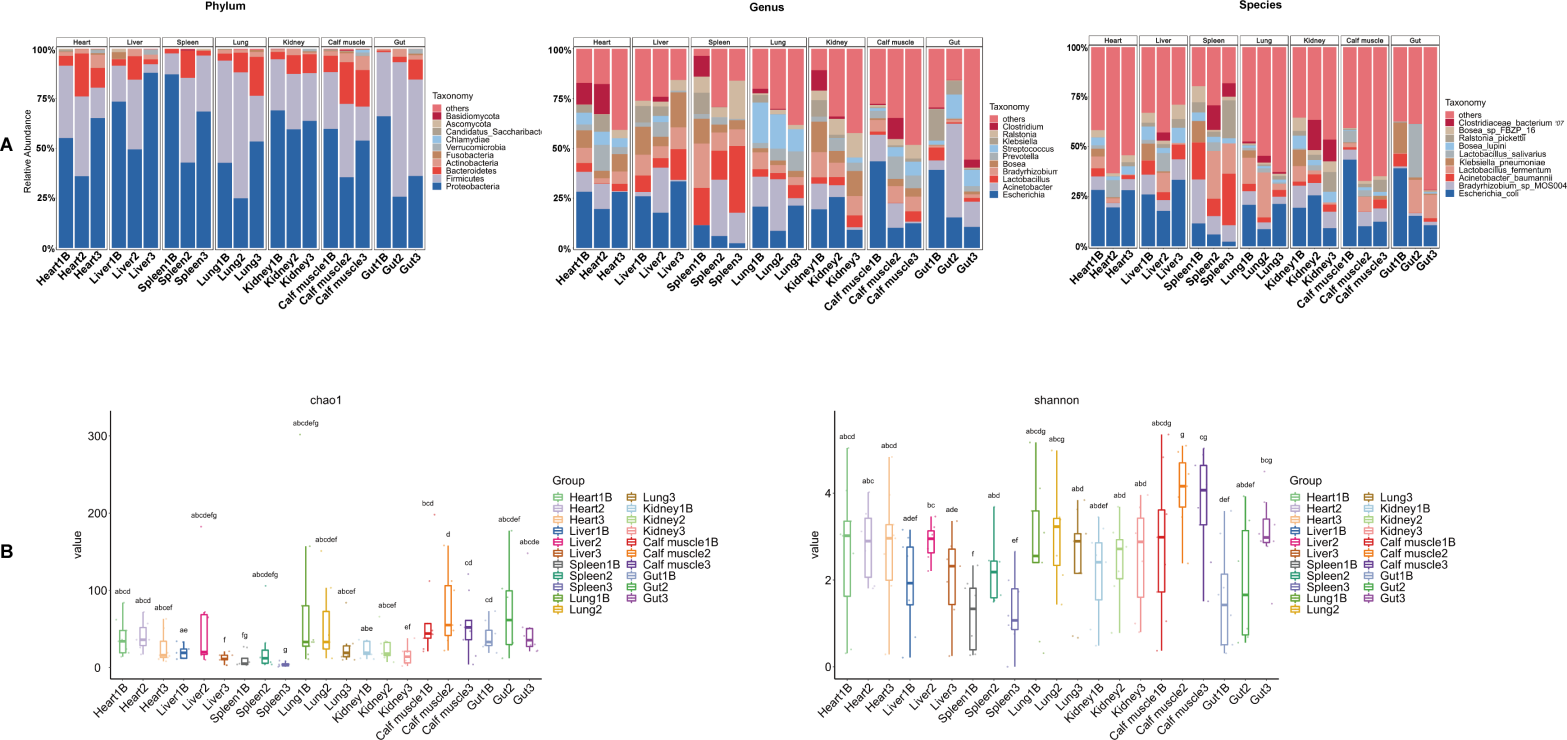
Overview of microbial community composition and alpha-diversity metrics. (**A**) Relative abundances of the 10 most abundant taxa at the phylum, genus, and species levels. (**B**) Features a box plot detailing the Chao1 and Shannon diversity indices. The same letter denoted above two groups indicates there is no significant difference between them. Conversely, significant differences between them do exist.

We analyzed the microbial community across multiple organs and PMIs at the phylum, genus, and species levels. The thanatomicrobiome primarily clustered into three major phyla: *Proteobacteria*, *Firmicutes*, and *Bacteroidetes*. Among these, *Proteobacteria* (25%–88% relative abundance) and *Firmicutes* (4%–68% relative abundance) were the most dominant across the samples (Fig. 2A). *Proteobacteria* showed a dynamic pattern, decreasing initially and then increasing with prolonged PMI, whereas *Firmicutes* followed an opposite trend.

At the genus level, *Escherichia* was the most prevalent taxon in many organ samples, followed by *Lactobacillus* (Fig. 2A). Tissue-specific microbial profiles were evident. Spleen samples contained elevated *Acinetobacter* (19%–34%) and variable levels of *Lactobacillus* (0.1%–28%). Heart samples showed consistently high levels of *Escherichia* (20%–28%). Lungs had higher abundances of *Streptococcus* (approximately 11%–20%), which decreased as PMI increased.

At the species level, *Escherichia coli* was the most common, with relative abundances of approximately 20%–28% in the heart, 18%–33% in the liver, 9%–26% in the kidney, 10%–43% in calf muscle, and 11%–39% in the gut (Fig. 2A). The facultative anaerobic nature of *E. coli* allows it to thrive in both aerobic and anaerobic conditions, which may explain its persistence after death. The spleen samples displayed variation in dominant species across PMI stages: Spleen1B (PMI 1–7 days) was dominated by *Acinetobacter baumannii* and *Bradyrhizobium* sp. MOS004; Spleen2 (PMI 9–12 days) by *Lactobacillus fermentum* and *Bradyrhizobium* sp. MOS004; and Spleen3 (PMI 15–31 days) by *Acinetobacter baumannii* and *Lactobacillus fermentum*. These findings highlight the potential forensic value of specific microbial signatures in PMI estimation.

### Changes in microbial diversity with increasing PMI

To assess microbial succession after death, we analyzed the alpha-diversity of microbial communities in different visceral organs across various PMIs using Chao1, Shannon, and Simpson indices.

The Chao1 index revealed significantly higher richness in calf muscle (mean Chao1: 64 ± 46) and gut samples (mean Chao1: 53 ± 42) compared to other tissues (*P* < 0.05, *t*-test) (Fig. 2B), reflecting the nutrient-rich and structurally complex environments of these organs. Following death, alpha-diversity trends varied noticeably across different tissues. For example, diversity in the lung and kidney declined with PMI, while other tissues displayed transient increase followed by decrease.

The Shannon index was lowest in spleen samples (mean Shannon index: 1.51 ± 0.87), suggesting a community dominated by a few species, linked to selective pressures from immune-related processes and nutrient influx during decomposition. In contrast, other tissues exhibited a more balanced microbial community, as indicated by more evenly distributed Shannon index values. Gut samples showed significant increases in Shannon diversity with prolonged PMI, while liver and spleen samples displayed elevated diversity in group 2 (PMI 9–12 days) compared with group 1B (PMI 1–7 days), followed by a decline in group 3 (PMI 15–31 days).

Taken together, these findings reveal dynamic, organ-specific patterns of microbial succession after death, highlighting the importance of organ context in postmortem microbiome analysis and the forensic potential of such signatures in PMI estimation.

### Comparison of samples from frozen and unfrozen cadavers

In our study, tissues and organs were collected from 32 cadavers, of which 11 were sampled fresh and 21 were stored at −15 to −20°C within 2 h postmortem before dissection. To evaluate the potential impact of freezing on the microbiota, we compared microbial communities from samples with a PMI of 1–7 days between unfrozen cadavers (group 1A) and frozen cadavers (group 1B).

**Fig 3:**
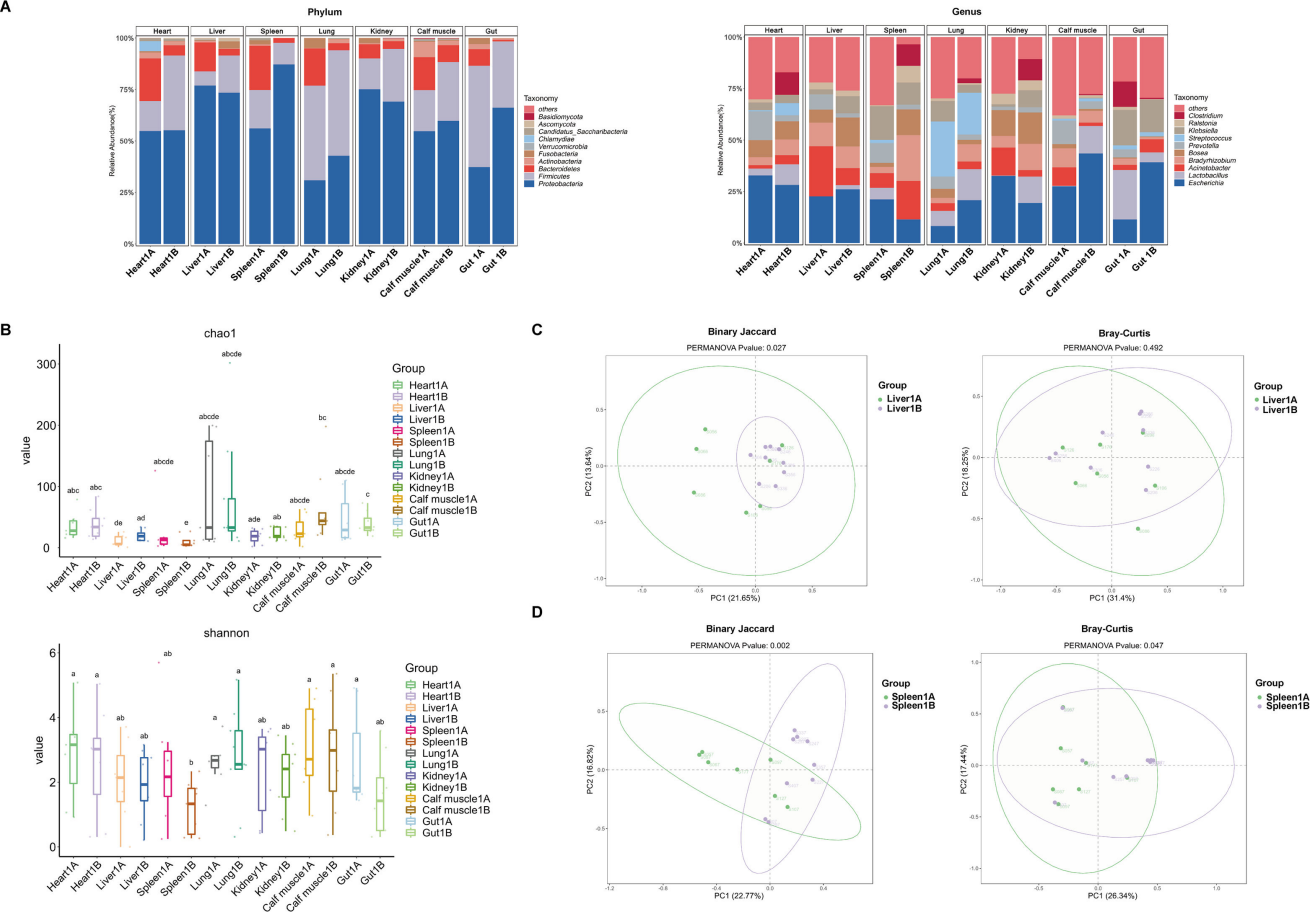
Impact of freezing on microbial communities of cadavers. (**A**) Relative abundances of the 10 most abundant taxa at the phylum and genus levels. (**B**) Features a box plot detailing the Chao1 and Shannon diversity indices. The same letter denoted above two groups indicates there is no significant difference between them. Conversely, significant differences between them do exist. (**C**) PCoA based on Bray-Curtis and Jaccard dissimilarity matrices of liver microbiota. (**D**) PCoA based on Bray-Curtis and Jaccard dissimilarity matrices of spleen microbiota. Samples are colored by their freezing status.

At the phylum level, *Proteobacteria*, *Firmicutes,* and *Bacteroidetes* were the dominant taxa across all samples (Fig. 3A). Unfrozen samples showed a higher relative abundance of *Bacteroidetes*, while *Firmicutes* were generally less abundant except in spleen and gut tissues. At the genus level, unfrozen samples were dominated by *Escherichia* and *Acinetobacter*. Freezing altered these compositions considerably, with *Escherichia* and *Acinetobacte*r declining in most frozen samples. Intriguingly, *Clostridium,* which was either absent or rare in unfrozen organs, became a predominant genus in frozen heart, spleen, lung, and kidney samples. Alpha-diversity analysis showed that the frozen liver (Liver1B) exhibited a significantly higher Chao1 index compared with the unfrozen liver (Liver1A) (Fig. 3B), indicating greater species richness in frozen cadavers. No significant differences in the Chao1 index were observed between frozen and unfrozen groups for the other tissues. The Shannon indices did not differ significantly between frozen and unfrozen states for any tissue.

We performed beta-diversity analyses to evaluate the effect of freezing preservation on microbial communities using samples with a PMI of 1–7 days. PCoA based on Bray-Curtis (quantitative) and Jaccard (binary) distances revealed that the microbial community composition was significantly altered by freezing in the liver and the spleen (PERMANOVA, *P* < 0.05; Fig. 3C and D). Notably, in the liver, separation between frozen and unfrozen samples was significant only with Jaccard (presence/absence) distances (Fig. 3C).

### Microbial abundance changes with PMI

**Fig 4:**
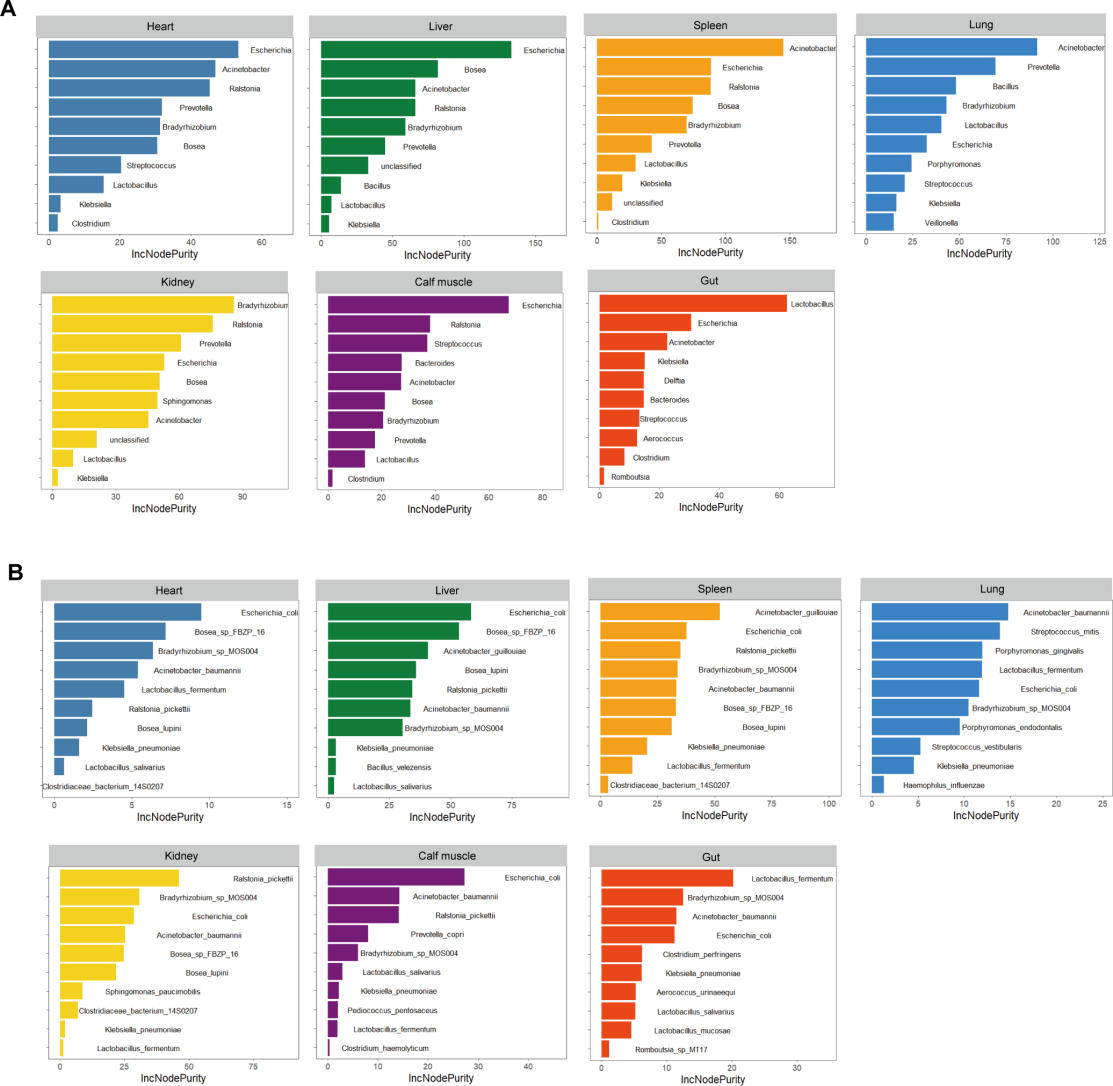
The microbial genera and species associated with PMI in each organ. (**A**) At the genus level. (**B**) At the species level.

To identify biomarker taxa and evaluate their relationship with PMI in various organs, we employed a random forest algorithm. The importance of each variable was assessed using the “IncNodePurity” metric, where higher values indicate a stronger correlation between a species and PMI. The top 10 bacterial taxa at the genus and species levels, ranked by their time-discriminatory importance during decomposition, are displayed in Fig. 4.

At the genus level, *Escherichia*, *Acinetobacter*, and *Lactobacillus* were consistently associated with PMI across organs. Specifically, *Escherichia* had the strongest correlation with PMI in the heart, liver, and calf muscle. In contrast, *Acinetobacter* was most relevant in the spleen and lung, *Lactobacillus* in the gut, and *Bradyrhizobium* in the spleen (Fig. 4A). At the species level, *Escherichia coli* showed the highest correlation with PMI in the heart, liver, and calf muscle (Fig. 4B), mirroring the genus-level findings. This suggests that changes in *Escherichia coli* abundance were most pronounced in these tissues as decomposition progressed. Other organ-specific associations included *Acinetobacter guillouiae* (spleen), *Acinetobacter baumannii* (lung), *Ralstonia pickettii* (kidney), and *Lactobacillus fermentum* (gut).

**Fig 5:**
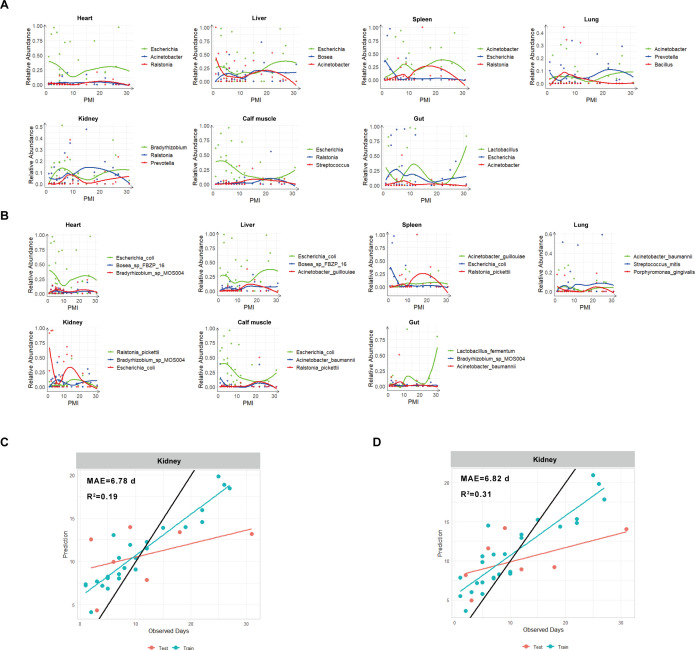
The changes of the three most important microbial taxa in each organ sample and the random forest graph for kidney samples. (**A**) The changes of the three most important microbial genera in each organ sample with PMI. (**B**) The changes of the three most important microbial species in each organ sample with PMI. (**C**) Random forest genus-level graph for spleen samples. (**D**) Random forest species-level graph for spleen samples.

Further analysis of the relative abundance trends of the three most crucial microbial genera and species associated with PMI in each organ revealed complex patterns of variation (Fig. 5A and B). Notably, most genera exhibited significant shifts around day 10. This stage marks a transition from anaerobic to aerobic conditions following the rupture of the body, leading to drastic changes in community composition.

At the genus level (Fig. 5A), *Prevotella* in kidney samples increased during the first 10 days, decreased between days 10 and 15, and rose again from days 15 to 30. In the gut, *Escherichia* initially increased and then decreased within the first 10 days, while *Lactobacillus* exhibited the opposite trend. In the spleen, *Escherichia* and *Acinetobacter* displayed complementary patterns.

At the species level (Fig. 5B), the early postmortem period was dominated by aerobic bacteria such as *Escherichia coli*, which initially decreased and then increased with prolonged PMI across most organs, except for the gut and spleen. In the spleen, *Escherichia coli* was predominant in the early postmortem period, while *Ralstonia pickettii* dominated the later stages. The gut microbiota showed distinct differences, particularly at a PMI of 20–31 days, where *Lactobacillus fermentum* significantly increased in relative abundance.

### Machine learning for PMI prediction

To evaluate the predictive utility of microbial profiles, we developed a random forest regression model for PMI estimation, applying fivefold cross-validation to assess the significance of various bacterial families. Error rates varied depending on the number of taxa included (Fig. S1). For example, in heart samples, error was highest when only three genera were included and lowest with 38 key genera. Similarly, in liver samples, minimal error was observed with 40 important genera. We determined the optimal number of microbial genera or species for each organ’s model based on these cross-validation results.

At the genus level (Fig. S2A), the gut model had the highest prediction error, with an MAE of 8.86 days. In contrast, the heart model showed the smallest error, with an MAE of 6.26 days, although it had relatively low accuracy (*R*^2^ = 0.13). The kidney model demonstrated the best predictive performance, with an MAE of 6.78 days and an *R*^2^ value of 0.19 (Fig. 5C).

At the species level, performance improved for all tissues except the liver (Fig. S2B). The heart model again exhibited the smallest error, with the MAE decreasing to 5.66 days and the *R*^2^ increasing to 0.21. Conversely, the calf muscle model performed worst, with an MAE of 8.28 days. The kidney model displayed a slightly higher MAE of 6.82 days, but its *R*^2^ improved to 0.31 (Fig. 5D).

## DISCUSSION

Our study provides novel insights into postmortem microbial succession by examining communities across multiple internal organs, calf muscles, and gut tissues in human cadavers from real forensic cases. This approach distinguishes our work from previous research that primarily centered on model organisms (30) (e.g., mice and swine) or human skin. Following the cessation of the immune function, human tissues become fertile grounds for microbial proliferation, particularly from intestinal and respiratory origins (31). These processes highlight the critical role of microbial metabolism in decomposition, where aerobic bacteria initially consume available oxygen, creating anaerobic conditions that favor the growth of intestinal taxa (11, 32). Notably, the cadaver samples in this study were not subjected to burial or similar processes, minimizing environmental influence such as insect activity or soil contamination (15, 33), and providing clearer insights into intrinsic succession of postmortem microbes.

At the phylum level, we found that *Proteobacteria* dominated early postmortem intervals, whereas *Firmicutes* and *Bacteroidetes* increased with advancing PMI. This transition reflects the shift from aerobic to anaerobic conditions during decomposition. However, these patterns varied across tissues and time points, reflecting the complexity of microbial dynamics. These findings are consistent with previous reports documenting similar trends in human decomposition (34).

We also compared postmortem microbiota between frozen and unfrozen cadavers sampled within 1–7 days PMI. Freezing altered the relative abundance of certain genera, such as *Clostridium*. In unfrozen organs, *Clostridium* was undetectable or minimal, but it emerged as a dominant genus in frozen heart, spleen, lung, and kidney samples. As a strict anaerobe capable of sporulation, *Clostridium* exhibits high resistance to environmental stresses such as freezing. Freezing appears to selectively inhibit more sensitive genera (e.g., *Escherichia* and *Acinetobacter*), thereby creating ecological opportunities for stress-tolerant taxa (such as *Clostridium*) to proliferate, ultimately altering the compositional structure of the cadaver microbiome.

Beta-diversity analyses revealed that freezing significantly affected the overall microbiota composition only in the liver and spleen. This limited effect may be attributable to the small sample size per group and the large within-group differences resulting from high individual variability. In addition, the PMIs we analyzed were short (1–7 days), and the non-frozen samples were concentrated in March when room temperature was relatively low, so microbial changes were not pronounced. In liver samples, PCoA using Bray-Curtis dissimilarity showed no significant separation between frozen and non-frozen groups, indicating that the dominant microbial taxa were largely unaffected under different storage conditions. In contrast, Jaccard-based PCoA revealed clear differences, indicating that freezing significantly influenced the presence or absence of low-abundance or rare taxa rather than the core microbiome. These findings imply that freezing mainly affected the rare biosphere while leaving the core decomposer community largely intact. For forensic studies, this suggests that reliance on low-abundance taxa as biomarkers may lead to distorted interpretations, while focusing on prevalent microbial taxa offers more consistent and reliable results. Future research using controlled animal models (e.g., pigs and rats) will allow freeze–thaw cycles to be systematically evaluated under specific conditions to better understand their effects on microbial succession.

The nonlinear relationship between microbial abundance and PMI across different time periods highlights the complexity of decomposition. For example, *Lactobacillus,* a facultative anaerobe common to the gastrointestinal tract, was more abundant in shorter PMIs in both our study and previous reports (5). In our study, *Lactobacillus* abundance increased significantly in multiple tissues at 9–12 days PMI, likely reflecting microbial translocation from the gut as the intestinal barrier breaks down. Likewise, *Clostridium* was predominantly detected in group 1B (PMI 1–7 days, frozen) and group 2 (PMI 9–12 days), consistent with previous reports of extremely rapid postmortem overgrowth in decaying internal sites such as blood, bone marrow, and liver (3, 5). Notably, *Clostridium* was absent from non-frozen group 1A (PMI 1–7 days, room temperature) tissues except the gut, a finding likely influenced by sample size and substantial individual variability. Collectively, these findings underscore the predictable changes in microbial composition within internal organs over time, with implications for PMI estimation.

Gradual changes in microbial communities after death can be used to determine PMI. Due to the challenges in obtaining human cadaver samples, research in this area has primarily focused on model organisms such as mice and swine (13, 16, 35). For instance, Metcalf et al. showed that PMI can be estimated within approximately 3 days over 48 days in a mouse model (36). While the microbial succession in these model organisms shares similarities with that in humans, notable differences exist that limit the direct applicability of these findings to human cases. Our study is distinctive in that all samples were derived from human cadavers (*n* = 32), representing a relatively large cohort compared to previous work. Prior PMI prediction models have predominantly utilized samples from human guts, skin, or soil surrounding cadavers, with limited studies focusing on internal organs. In contrast, we analyzed tissues from multiple organs, including heart, liver, spleen, lungs, kidneys, calf muscles, and guts. Among these, the kidney model exhibited the best performance, with an error of 6.82 days over a 30 day window. Although the MAE of the sample was relatively high, it was based on the data of human samples. Existing PMI prediction models constructed using human samples typically exhibit lower accuracy. For example, Lai Hu et al. reported an appendix-based model with an MAE of 25.79 ± 0.43 h during 192 h of decomposition, though their sample size was small and the data set was not divided into training and testing sets (14).

One limitation of our study is that frozen and non-frozen samples were combined (*n* = 32) to strengthen the statistical power of PMI modeling. Although this allows us to capture general microbial successional patterns, freezing and storage can alter community composition, potentially introducing bias. Consequently, the predictive performance of our model may be reduced when applied to unpreserved remains. Future studies with larger cohorts of fresh samples, ideally collected through standardized forensic protocols, will be essential to validate and refine PMI estimation models and to disentangle storage-related effects from true postmortem microbial dynamics.

Our study highlights the limitations of the random forest regression model used to estimate PMI, which underscores the inherent complexity of decomposition processes. The nonuniform changes in *Escherichia* abundance, particularly in cardiac tissues, exemplify the dynamic and nonlinear nature of microbial succession, posing challenges for the development of universal predictive models. Microbial community dynamics are highly variable across different organs, emphasizing the need for stage-specific models (37) that capture the nuances of microbial activity at various decomposition phases. Environmental factors such as temperature, humidity, and soil type further complicate the modeling by significantly influencing microbial communities and decomposition rates (28). These variables can introduce variability that standard models may not fully account for, potentially leading to inaccuracies in PMI estimation. Additionally, individual differences, including age, cause of death, and overall health, add another layer of variability (38, 39). Although some studies have shown consistent microbial networks despite environmental variations (15), individual-specific factors remain a significant obstacle to PMI prediction.

To address these challenges, future research should integrate environmental factors, individual cadaver characteristics, storage conditions, and microbial community dynamics into advanced modeling techniques. Larger and more diverse human cadaver data sets will enable the construction of more robust and generalizable models. Such models would not only enhance PMI prediction accuracy but also improve forensic applicability across varied contexts.

The forensic utility of microbiological data lies in its potential to complement traditional PMI estimation methods. In situations where entomological or physiological indicators (40) are unavailable or compromised, microbial succession provides an independent and valuable line of evidence. By identifying tissue-specific microbial signatures associated with decomposition stages, investigators can refine PMI estimates and strengthen forensic interpretations.

Finally, our findings emphasize the critical need for standardization in forensic microbiology research. Developing consistent protocols that consider environmental variables and individual differences will be essential to enhance reproducibility of microbial-based PMI estimations. Collaborative efforts to establish reference data sets and harmonized methodologies will improve the accuracy and reliability of forensic investigations.

### Conclusions

This study provides a comprehensive examination of postmortem microbial succession across various human organs, including the heart, liver, spleen, lungs, kidneys, calf muscles, and gut, using samples from 32 cadavers. It represents the first application of 2bRAD-M sequencing technology for detecting postmortem microbes across a broad range of human tissues. Our findings demonstrate that 2bRAD-M technology accurately and sensitively detects microbes, even in significantly degraded samples, underscoring its suitability for thanatomicrobiome research.

Our results reveal significant variability in microbial community composition across different organs and PMIs. The nonlinear trends observed in microbial succession highlight the complexity of decomposition processes. The comparison between frozen and unfrozen cadavers revealed minimal impact of freezing on most tissues, except for the liver and kidneys. Additionally, environmental conditions and individual circumstances further influence decomposition, which may contribute to the limitations of current PMI prediction models. Future research should explore these factors in greater depth.

In summary, this study advances our understanding of microbial succession in multiple human organs after death. Investigating the pathways and mechanisms that drive postmortem microbial changes is crucial for improving PMI estimation methods and enhancing our overall understanding of the decomposition process.

## Data Availability

The relevant data used to build the random forest models are included as Additional files 2–15, respectively. Original R scripts are available in GitHub (https://github.com/zengjianye1/Random-Forest). All raw sequencing data generated during the current study are available in the NCBI Sequence Read Archive (SRA): PRJNA1165987.

## SUPPLEMENTAL MATERIAL

Fig. S1 spectrum.02666-24-s0001.tif Cross-validation curves of various organ samples.

Fig. S2 spectrum.02666-24-s0002.tif PMI prediction models based on multiple tissue types.

Supplemental material spectrum.02666-24-s0003.docx Legends for Supplemental figures and tables.

Table S1 spectrum.02666-24-s0004.docx Sample information of 32 cadavers in this study.

Table S2 spectrum.02666-24-s0005.docx Data summary table.

ASM does not own the copyrights to Supplemental Material that may be linked to, or accessed through, an article. The authors have granted ASM a non-exclusive, world-wide license to publish the Supplemental Material files. Please contact the corresponding author directly for reuse.

## References

[1] Turnbaugh PJ, Ley RE, Hamady M, Fraser-Liggett C, Knight R, Gordon JI. 2007. The human microbiome project: exploring the microbial part of ourselves in a changing world. Nature 449:804–810. doi:10.1038/nature06244 17943116 PMC3709439

[2] Martino C, Dilmore AH, Burcham ZM, Metcalf JL, Jeste D, Knight R. 2022. Microbiota succession throughout life from the cradle to the grave. Nat Rev Microbiol 20:707–720. doi:10.1038/s41579-022-00768-z 35906422 PMC12875531

[3] Javan GT, Finley SJ, Abidin Z, Mulle JG. 2016. The thanatomicrobiome: a missing piece of the microbial puzzle of death. Front Microbiol 7:225. doi:10.3389/fmicb.2016.00225 26941736 PMC4764706

[4] Paczkowski S, Schütz S. 2011. Post-mortem volatiles of vertebrate tissue. Appl Microbiol Biotechnol 91:917–935. doi:10.1007/s00253-011-3417-x 21720824 PMC3145088

[5] Can I, Javan GT, Pozhitkov AE, Noble PA. 2014. Distinctive thanatomicrobiome signatures found in the blood and internal organs of humans. J Microbiol Methods 106:1–7. doi:10.1016/j.mimet.2014.07.026 25091187

[6] Lee Goff M. 2009. Early post-mortem changes and stages of decomposition in exposed cadavers. Exp Appl Acarol 49:21–36. doi:10.1007/s10493-009-9284-9 19554461

[7] Hyde ER, Haarmann DP, Lynne AM, Bucheli SR, Petrosino JF. 2013. The living dead: bacterial community structure of a cadaver at the onset and end of the bloat stage of decomposition. PLoS One 8:e77733. doi:10.1371/journal.pone.0077733 24204941 PMC3813760

[8] Metcalf JL. 2019. Estimating the postmortem interval using microbes: knowledge gaps and a path to technology adoption. Forensic Sci Int Genet 38:211–218. doi:10.1016/j.fsigen.2018.11.004 30448529

[9] DeBruyn JM, Hauther KA. 2017. Postmortem succession of gut microbial communities in deceased human subjects. PeerJ 5:e3437. doi:10.7717/peerj.3437 28626612 PMC5470579

[10] Hauther KA, Cobaugh KL, Jantz LM, Sparer TE, DeBruyn JM. 2015. Estimating time since death from postmortem human gut microbial communities. J Forensic Sci 60:1234–1240. doi:10.1111/1556-4029.12828 26096156

[11] Heimesaat MM, Boelke S, Fischer A, Haag L-M, Loddenkemper C, Kühl AA, Göbel UB, Bereswill S. 2012. Comprehensive postmortem analyses of intestinal microbiota changes and bacterial translocation in human flora associated mice. PLoS One 7:e40758. doi:10.1371/journal.pone.0040758 22808253 PMC3395637

[12] Javan GT, Finley SJ. 2018. Chapter 6 - what is the “thanatomicrobiome” and what is its relevance to forensic investigations, p 133–143. In Ralebitso-Senior TK (ed), Forensic Ecogenomics. Academic Press.

[13] Liu R, Gu Y, Shen M, Li H, Zhang K, Wang Q, Wei X, Zhang H, Wu D, Yu K, Cai W, Wang G, Zhang S, Sun Q, Huang P, Wang Z. 2020. Predicting postmortem interval based on microbial community sequences and machine learning algorithms. Environ Microbiol 22:2273–2291. doi:10.1111/1462-2920.15000 32227435

[14] Hu L, Xing Y, Jiang P, Gan L, Zhao F, Peng W, Li W, Tong Y, Deng S. 2021. Predicting the postmortem interval using human intestinal microbiome data and random forest algorithm. Sci Justice 61:516–527. doi:10.1016/j.scijus.2021.06.006 34482931

[15] Burcham ZM, Belk AD, McGivern BB, Bouslimani A, Ghadermazi P, Martino C, Shenhav L, Zhang AR, Shi P, Emmons A, et al.. 2024. A conserved interdomain microbial network underpins cadaver decomposition despite environmental variables. Nat Microbiol 9:595–613. doi:10.1038/s41564-023-01580-y 38347104 PMC10914610

[16] Pechal JL, Crippen TL, Benbow ME, Tarone AM, Dowd S, Tomberlin JK. 2014. The potential use of bacterial community succession in forensics as described by high throughput metagenomic sequencing. Int J Legal Med 128:193–205. doi:10.1007/s00414-013-0872-1 23749255

[17] Stokes KL, Forbes SL, Tibbett M. 2013. Human versus animal: contrasting decomposition dynamics of mammalian analogues in experimental taphonomy. J Forensic Sci 58:583–591. doi:10.1111/1556-4029.12115 23550805

[18] Lamendella R, Santo Domingo JW, Ghosh S, Martinson J, Oerther DB. 2011. Comparative fecal metagenomics unveils unique functional capacity of the swine gut. BMC Microbiol 11:103. doi:10.1186/1471-2180-11-103 21575148 PMC3123192

[19] Pechal JL, Schmidt CJ, Jordan HR, Benbow ME. 2018. A large-scale survey of the postmortem human microbiome, and its potential to provide insight into the living health condition. Sci Rep 8:5724. doi:10.1038/s41598-018-23989-w 29636512 PMC5893548

[20] Javan GT, Finley SJ, Can I, Wilkinson JE, Hanson JD, Tarone AM. 2016. Human thanatomicrobiome succession and time since death. Sci Rep 6:29598. doi:10.1038/srep29598 27412051 PMC4944132

[21] Ranjan R, Rani A, Metwally A, McGee HS, Perkins DL. 2016. Analysis of the microbiome: advantages of whole genome shotgun versus 16S amplicon sequencing. Biochem Biophys Res Commun 469:967–977. doi:10.1016/j.bbrc.2015.12.083 26718401 PMC4830092

[22] Shi Y, Wang G, Lau H-H, Yu J. 2022. Metagenomic sequencing for microbial DNA in human samples: emerging technological advances. IJMS 23:2181. doi:10.3390/ijms23042181 35216302 PMC8877284

[23] Sun Z, Huang S, Zhu P, Tzehau L, Zhao H, Lv J, Zhang R, Zhou L, Niu Q, Wang X, Zhang M, Jing G, Bao Z, Liu J, Wang S, Xu J. 2022. Species-resolved sequencing of low-biomass or degraded microbiomes using 2bRAD-M. Genome Biol 23:36. doi:10.1186/s13059-021-02576-9 35078506 PMC8789378

[24] Huang X, Zeng J, Li S, Chen J, Wang H, Li C, Zhang S. 2024. 16S rRNA, metagenomics and 2bRAD-M sequencing to decode human thanatomicrobiome. Sci Data 11:736. doi:10.1038/s41597-024-03518-3 38971804 PMC11227556

[25] Martin M. 2011. Cutadapt removes adapter sequences from high-throughput sequencing reads. EMBnet j 17:10. doi:10.14806/ej.17.1.200

[26] Langmead B, Salzberg SL. 2012. Fast gapped-read alignment with Bowtie 2. Nat Methods 9:357–359. doi:10.1038/nmeth.1923 22388286 PMC3322381

[27] Bolyen E, Rideout JR, Dillon MR, Bokulich NA, Abnet CC, Al-Ghalith GA, Alexander H, Alm EJ, Arumugam M, Asnicar F, et al.. 2019. Reproducible, interactive, scalable and extensible microbiome data science using QIIME 2. Nat Biotechnol 37:852–857. doi:10.1038/s41587-019-0209-9 31341288 PMC7015180

[28] Chao A, Bunge J. 2002. Estimating the number of species in a stochastic abundance model. Biometrics 58:531–539. doi:10.1111/j.0006-341X.2002.00531.x 12229987

[29] Hill T. 2003. Using ecological diversity measures with bacterial communities. FEMS Microbiol Ecol 43:1–11. doi:10.1016/S0168-6496(02)00449-X 19719691

[30] Ferreira PG, Muñoz-Aguirre M, Reverter F, Sá Godinho CP, Sousa A, Amadoz A, Sodaei R, Hidalgo MR, Pervouchine D, Carbonell-Caballero J, Nurtdinov R, Breschi A, Amador R, Oliveira P, Çubuk C, Curado J, Aguet F, Oliveira C, Dopazo J, Sammeth M, Ardlie KG, Guigó R. 2018. The effects of death and post-mortem cold ischemia on human tissue transcriptomes. Nat Commun 9:490. doi:10.1038/s41467-017-02772-x 29440659 PMC5811508

[31] Dent BB, Forbes SL, Stuart BH. 2004. Review of human decomposition processes in soil. Environmental Geology 45:576–585. doi:10.1007/s00254-003-0913-z

[32] Burcham ZM, Hood JA, Pechal JL, Krausz KL, Bose JL, Schmidt CJ, Benbow ME, Jordan HR. 2016. Fluorescently labeled bacteria provide insight on post-mortem microbial transmigration. Forensic Sci Int 264:63–69. doi:10.1016/j.forsciint.2016.03.019 27032615

[33] Campobasso CP, Di Vella G, Introna F. 2001. Factors affecting decomposition and Diptera colonization. Forensic Sci Int 120:18–27. doi:10.1016/S0379-0738(01)00411-X 11457604

[34] Zhang Y, Pechal JL, Schmidt CJ, Jordan HR, Wang WW, Benbow ME, Sze S-H, Tarone AM. 2019. Machine learning performance in a microbial molecular autopsy context: a cross-sectional postmortem human population study. PLoS ONE 14:e0213829. doi:10.1371/journal.pone.0213829 30986212 PMC6464165

[35] Carter DO, Metcalf JL, Bibat A, Knight R. 2015. Seasonal variation of postmortem microbial communities. Forensic Sci Med Pathol 11:202–207. doi:10.1007/s12024-015-9667-7 25737335 PMC9636889

[36] Metcalf JL, Wegener Parfrey L, Gonzalez A, Lauber CL, Knights D, Ackermann G, Humphrey GC, Gebert MJ, Van Treuren W, Berg-Lyons D, Keepers K, Guo Y, Bullard J, Fierer N, Carter DO, Knight R. 2013. A microbial clock provides an accurate estimate of the postmortem interval in a mouse model system. Elife 2:e01104. doi:10.7554/eLife.01104 24137541 PMC3796315

[37] Li N, Liang X-R, Zhou S-D, Dang L-H, Li J, An G-S, Ren K, Jin Q-Q, Liang X-H, Cao J, Du Q-X, Wang Y-Y, Sun J-H. 2023. Exploring postmortem succession of rat intestinal microbiome for PMI based on machine learning algorithms and potential use for humans. Forensic Sci Int Genet 66:102904. doi:10.1016/j.fsigen.2023.102904 37307769

[38] Dash HR, Das S. 2020. Thanatomicrobiome and epinecrotic community signatures for estimation of post-mortem time interval in human cadaver. Appl Microbiol Biotechnol 104:9497–9512. doi:10.1007/s00253-020-10922-3 33001249

[39] Javan GT, Finley SJ, Tuomisto S, Hall A, Benbow ME, Mills D. 2019. An interdisciplinary review of the thanatomicrobiome in human decomposition. Forensic Sci Med Pathol 15:75–83. doi:10.1007/s12024-018-0061-0 30519986

[40] Henssge C, Madea B. 2004. Estimation of the time since death in the early post-mortem period. Forensic Sci Int 144:167–175. doi:10.1016/j.forsciint.2004.04.051 15364387

