# Supplementary figures and images for: Microbial succession in human tissues postmortem: insights from 2bRAD-M sequencing
Source: Microbiol Spectr. 2025 Nov 17;14(1):e02666-24. doi: 10.1128/spectrum.02666-24 (PMC12772320; doi:10.1128/spectrum.02666-24)

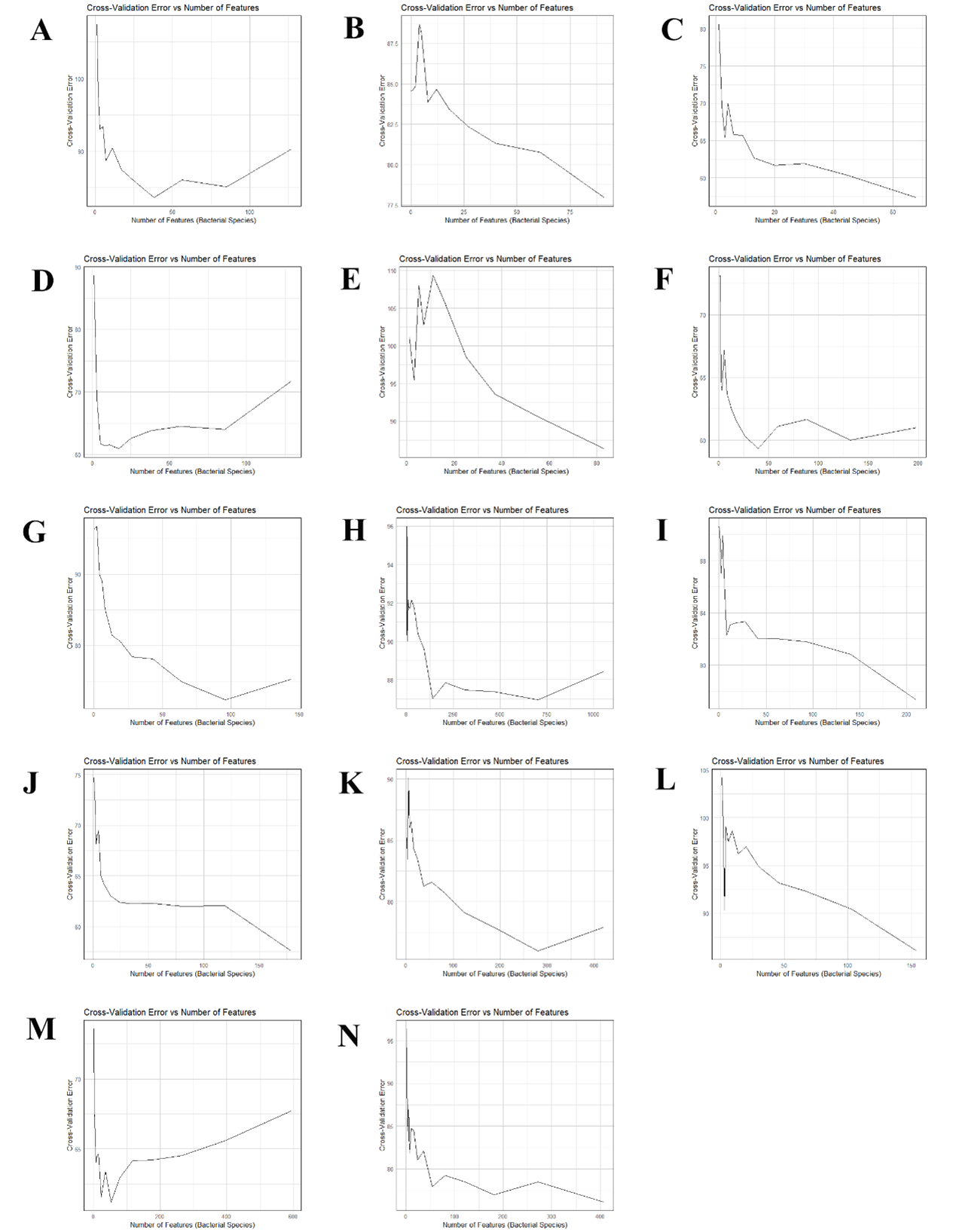

Supplement: Fig. S1 — Cross-validation curves of various organ samples. [file spectrum.02666-24-s0001.tif]

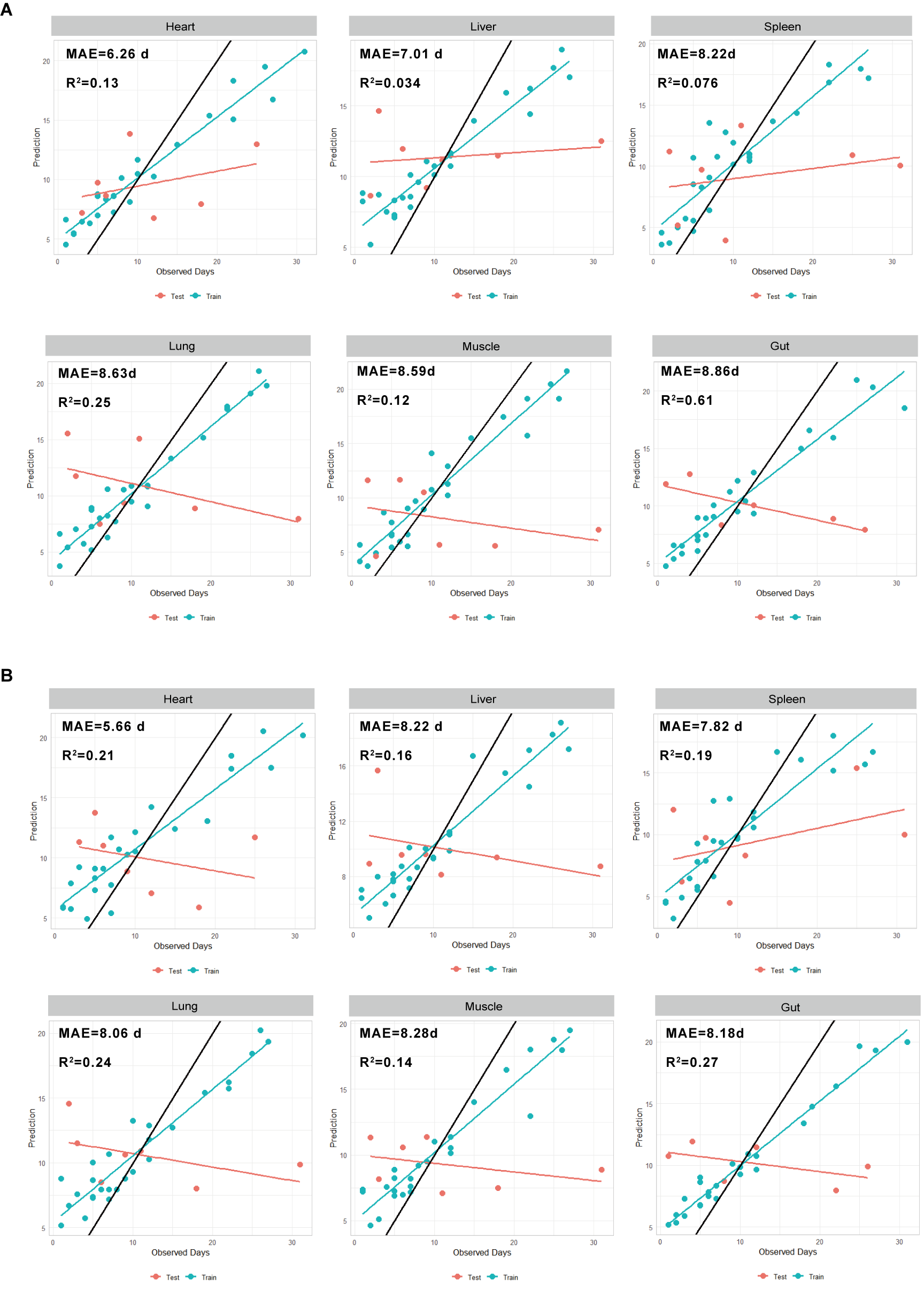

Supplement: Fig. S2 — PMI prediction models based on multiple tissue types. [file spectrum.02666-24-s0002.tif]
