# Supplementary material for: Microbial succession in human tissues postmortem: insights from 2bRAD-M sequencing
Source: Microbiol Spectr. 2025 Nov 17;14(1):e02666-24. doi: 10.1128/spectrum.02666-24 (PMC12772320; doi:10.1128/spectrum.02666-24)
Supplement: Supplemental material — Legends for Supplemental figures and tables. [file spectrum.02666-24-s0003.docx]

**SUPPLEMENTAL MATERIAL**

Supplementary Table 1

Sample information of 32 cadavers in this study

Supplementary Table 2

Data summary table

Fig S1 goes here

Supplementary figure 1. Cross-validation curves of various organ samples. (A) heart samples at the genus level (B) liver samples at the genus level (C) spleen samples at the genus level (D) lung samples at the genus level (E) kidney samples at the genus level (F) calf muscle samples at the genus level (G) gut samples at the genus level (H) heart samples at the species level (I) liver samples at the species level (J) spleen samples at the species level (K) lung samples at the species level (L) kidney samples at the species level (M) calf muscle samples at the species level (N) gut samples at the species level.

Fig S2 goes here

Supplementary figure 2. PMI prediction models based on multiple tissue types. (A) at the genus level (B) at the species level.
