# Supplementary material for: Microbial succession in human tissues postmortem: insights from 2bRAD-M sequencing
Source: Microbiol Spectr. 2025 Nov 17;14(1):e02666-24. doi: 10.1128/spectrum.02666-24 (PMC12772320; doi:10.1128/spectrum.02666-24)
Supplement: Table S1 — Sample information of 32 cadavers in this study. [file spectrum.02666-24-s0004.docx]

Supplementary Table 1

Sample information of 32 cadavers in this study

| **Group** | **Individual code** | **PMI（days）** | **Age (years)** | **Sex** | **Cause of Death** | **Date of Death (Y.M.D)** | **Sampling Date (Y.M.D)** | **Frozen** | **Freezing temperature(℃)** | **Freezing time（days）** |
| --- | --- | --- | --- | --- | --- | --- | --- | --- | --- | --- |
| 1 | S06 | 1 | 41 | M | Craniocerebral injury | 2023/3/6 | 2023/3/7 | No | - | - |
|  | S08 | 1 | 47 | M | Coronary heart disease | 2023/3/9 | 2023/3/10 | No | - | - |
|  | S09 | 2 | 37 | F | Craniocerebral injury | 2023/3/21 | 2023/3/23 | No | - | - |
|  | S10 | 2 | 48 | M | Coronary heart disease | 2023/3/26 | 2023/3/28 | No | - | - |
|  | S12 | 3 | 47 | M | Drowning | 2023/3/31 | 2023/4/3 | No | - | - |
|  | S17 | 4 | 49 | M | Coronary heart disease | 2023/4/22 | 2023/4/26 | No | - | - |
|  | S05 | 5 | 80 | M | Stomach tumor and lung infection | 2023/2/24 | 2023/3/1 | No | - | - |
| 2 | S40 | 3 | 60 | F | Drug intoxication | 2023/9/15 | 2023/9/18 | Yes | -15-20 | 3 |
|  | S24 | 5 | 65 | M | Coronary heart disease | 2023/5/27 | 2023/6/1 | Yes | -15-20 | 5 |
|  | S30 | 5 | 51 | M | Traffic injury | 2023/7/1 | 2023/7/6 | Yes | -15-20 | 5 |
|  | S22 | 6 | 27 | M | Sudden death | 2023/5/20 | 2023/5/26 | Yes | -15-20 | 6 |
|  | S33 | 6 | 42 | M | Electric shock | 2023/8/17 | 2023/8/23 | Yes | -15-20 | 6 |
|  | S20 | 7 | 54 | M | Hanging | 2023/5/11 | 2023/5/19 | Yes | -15-20 | 7 |
|  | S25 | 7 | 62 | F | Septic shock and multiple organ failure | 2023/5/31 | 2023/6/7 | Yes | -15-20 | 7 |
|  | S28 | 7 | 57 | M | Sudden death | 2023/6/9 | 2023/6/16 | Yes | -15-20 | 7 |
|  | S35 | 7 | 78 | M | Aortoclasia | 2023/8/29 | 2023/9/5 | Yes | -15-20 | 7 |
| 3 | S01 | 9 | 54 | M | Craniocerebral injury | 2022/12/7 | 2022/12/16 | Yes | -15-20 | 9 |
|  | S21 | 9 | 80 | M | Traffic injury | 2023/5/16 | 2023/5/25 | No | - | - |
|  | S23 | 10 | 71 | M | Traffic injury | 2023/5/20 | 2023/5/30 | Yes | -15-20 | 10 |
|  | S19 | 10 | 55 | M | Acute pulmonary embolism | 2023/5/2 | 2023/5/12 | No | - | - |
|  | S14 | 12 | 23 | F | Injury by fall from height | 2023/3/23 | 2023/4/4 | Yes | -15-20 | 12 |
|  | S18 | 12 | 51 | M | Traumatic shock | 2023/4/30 | 2023/5/12 | No | - | - |
|  | S38 | 12 | 51 | M | Unknown | 2023/8/31 | 2023/9/12 | Yes | -15-20 | 12 |
| 4 | S02 | 15 | 64 | M | Sudden cardiac death | 2022/12/1 | 2022/12/16 | Yes | -15-20 | 15 |
|  | S27 | 18 | 46 | M | Traffic injury | 2023/5/29 | 2023/6/16 | Yes | -15-20 | 18 |
|  | S32 | 19 | 45 | M | Traffic injury | 2023/7/29 | 2023/8/17 | Yes | -15-20 | 19 |
|  | S04 | 22 | 87 | F | Coronary heart disease | 2023/2/2 | 2023/2/24 | Yes | -15-20 | 22 |
|  | S07 | 22 | 65 | F | Coronary heart disease | 2023/2/15 | 2023/3/9 | Yes | -15-20 | 22 |
|  | S11 | 25 | 28 | M | Craniocerebral injury | 2023/3/5 | 2023/3/30 | Yes | -15-20 | 25 |
|  | S34 | 26 | 74 | M | Traffic injury | 2023/8/16 | 2023/9/11 | Yes | -15-20 | 26 |
|  | S39 | 27 | 42 | M | Trauma | 2023/8/22 | 2023/9/18 | Yes | -15-20 | 27 |
|  | S13 | 31 | 57 | M | Coronary heart disease | 2023/3/3 | 2023/4/4 | No | - | - |
