# Supplementary material for: Microbial succession in human tissues postmortem: insights from 2bRAD-M sequencing
Source: Microbiol Spectr. 2025 Nov 17;14(1):e02666-24. doi: 10.1128/spectrum.02666-24 (PMC12772320; doi:10.1128/spectrum.02666-24)
Supplement: Table S2 — Data summary table. [file spectrum.02666-24-s0005.docx]

Supplementary Table 2

Data Summary Table

| **Sample** | **Raw reads** | **Clean reads** | **Percent** |
| --- | --- | --- | --- |
| S237 | 7832644 | 7356564 | 93.92% |
| S285 | 5907613 | 5538449 | 93.75% |
| S406 | 6546569 | 6132081 | 93.67% |
| S307 | 7057387 | 6609137 | 93.65% |
| S217 | 9215764 | 8627025 | 93.61% |
| S197 | 7275432 | 6808143 | 93.58% |
| S309 | 5547053 | 5187012 | 93.51% |
| S227 | 7386208 | 6893999 | 93.34% |
| S259 | 6172110 | 5755871 | 93.26% |
| S238 | 7319579 | 6820419 | 93.18% |
| S328 | 7400147 | 6881468 | 92.99% |
| S187 | 7514940 | 6987650 | 92.98% |
| S258 | 7346780 | 6829923 | 92.96% |
| S398 | 7678664 | 7130110 | 92.86% |
| S358 | 8563573 | 7944543 | 92.77% |
| S1911 | 6038179 | 5599901 | 92.74% |
| S177 | 6935393 | 6431178 | 92.73% |
| S218 | 6926104 | 6422324 | 92.73% |
| S408 | 7399004 | 6857390 | 92.68% |
| S129 | 5007472 | 4638421 | 92.63% |
| S099 | 8668014 | 8020815 | 92.53% |
| S278 | 6834275 | 6323084 | 92.52% |
| S248 | 8233766 | 7614224 | 92.48% |
| S198 | 7040149 | 6508989 | 92.46% |
| S139 | 5339745 | 4934434 | 92.41% |
| S119 | 8380787 | 7738502 | 92.34% |
| S1411 | 7343315 | 6773218 | 92.24% |
| S337 | 6673058 | 6152422 | 92.20% |
| S329 | 6009621 | 5540023 | 92.19% |
| S2011 | 6432414 | 5923586 | 92.09% |
| S108 | 7241123 | 6665390 | 92.05% |
| S306 | 6583185 | 6055078 | 91.98% |
| S388 | 8186632 | 7518523 | 91.84% |
| S2710 | 6602718 | 6061257 | 91.80% |
| S228 | 7675038 | 7042366 | 91.76% |
| S196 | 9095018 | 8339343 | 91.69% |
| S058 | 6552382 | 6007593 | 91.69% |
| S289 | 7340359 | 6729206 | 91.67% |
| S348 | 7400880 | 6776279 | 91.56% |
| S026 | 6381783 | 5840930 | 91.53% |
| S118 | 7744119 | 7086873 | 91.51% |
| S349 | 5631655 | 5150847 | 91.46% |
| S127 | 8387854 | 7670287 | 91.45% |
| S326 | 7667398 | 7010310 | 91.43% |
| S048 | 7942070 | 7259125 | 91.40% |
| S107 | 7502150 | 6855248 | 91.38% |
| S097 | 7524129 | 6874523 | 91.37% |
| S109 | 7695582 | 7031747 | 91.37% |
| S279 | 6948843 | 6349229 | 91.37% |
| S347 | 6674798 | 6097475 | 91.35% |
| S209 | 6258301 | 5716632 | 91.34% |
| S189 | 6194907 | 5658163 | 91.34% |
| S1811 | 5711148 | 5209541 | 91.22% |
| S396 | 6187627 | 5641886 | 91.18% |
| S0910 | 6907073 | 6297713 | 91.18% |
| S305 | 6170186 | 5622794 | 91.13% |
| S117 | 6505791 | 5925038 | 91.07% |
| S357 | 7132571 | 6493313 | 91.04% |
| S075 | 6679525 | 6079218 | 91.01% |
| S275 | 5539852 | 5041167 | 91.00% |
| S068 | 5983931 | 5443726 | 90.97% |
| S1110 | 6249147 | 5683716 | 90.95% |
| S3410 | 4660802 | 4238768 | 90.95% |
| S0811 | 7567968 | 6883207 | 90.95% |
| S2211 | 6061816 | 5511137 | 90.92% |
| S208 | 7394248 | 6721248 | 90.90% |
| S359 | 7247176 | 6587408 | 90.90% |
| S219 | 5532756 | 5028962 | 90.89% |
| S3211 | 7938989 | 7212933 | 90.85% |
| S046 | 7197648 | 6536478 | 90.81% |
| S1211 | 5658773 | 5138684 | 90.81% |
| S249 | 6581590 | 5975132 | 90.79% |
| S339 | 6230009 | 5654735 | 90.77% |
| S207 | 7022624 | 6374034 | 90.76% |
| S257 | 6245431 | 5665447 | 90.71% |
| S179 | 5016193 | 4549538 | 90.70% |
| S0911 | 5340533 | 4843085 | 90.69% |
| S277 | 6319403 | 5730729 | 90.68% |
| S047 | 7354287 | 6666438 | 90.65% |
| S055 | 6753748 | 6117440 | 90.58% |
| S1010 | 6193664 | 5609840 | 90.57% |
| S308 | 7894636 | 7140687 | 90.45% |
| S287 | 5959490 | 5380640 | 90.29% |
| S199 | 5656112 | 5100994 | 90.19% |
| S239 | 6587947 | 5941834 | 90.19% |
| S389 | 6583162 | 5936329 | 90.17% |
| S076 | 5304533 | 4779689 | 90.11% |
| S077 | 6687000 | 6025303 | 90.10% |
| S225 | 5943186 | 5354275 | 90.09% |
| S2811 | 7375501 | 6644204 | 90.08% |
| S1310 | 7596017 | 6840720 | 90.06% |
| S355 | 7517508 | 6768449 | 90.04% |
| S128 | 8527956 | 7678474 | 90.04% |
| S338 | 8997118 | 8095487 | 89.98% |
| S345 | 6737273 | 6058308 | 89.92% |
| S138 | 8816998 | 7925579 | 89.89% |
| S226 | 5752625 | 5165009 | 89.79% |
| S3310 | 4533066 | 4067444 | 89.73% |
| S188 | 8666401 | 7774320 | 89.71% |
| S019 | 7740960 | 6942374 | 89.68% |
| S2411 | 6715642 | 6022671 | 89.68% |
| S065 | 6521690 | 5847528 | 89.66% |
| S137 | 6707558 | 6012686 | 89.64% |
| S079 | 7282855 | 6528222 | 89.64% |
| S0510 | 8409226 | 7538275 | 89.64% |
| S1111 | 6069926 | 5439296 | 89.61% |
| S395 | 5573056 | 4993621 | 89.60% |
| S178 | 8418975 | 7542081 | 89.58% |
| S016 | 6379294 | 5712404 | 89.55% |
| S325 | 6618524 | 5925291 | 89.53% |
| S1810 | 6320313 | 5658208 | 89.52% |
| S1011 | 5562010 | 4976819 | 89.48% |
| S247 | 6198842 | 5546139 | 89.47% |
| S2711 | 7601754 | 6801476 | 89.47% |
| S125 | 6917787 | 6187021 | 89.44% |
| S3210 | 7161132 | 6403776 | 89.42% |
| S1210 | 7867770 | 7034117 | 89.40% |
| S2111 | 7026276 | 6281069 | 89.39% |
| S3510 | 4879309 | 4360877 | 89.37% |
| S2511 | 7318125 | 6540560 | 89.37% |
| S085 | 7239090 | 6468571 | 89.36% |
| S195 | 5212911 | 4658252 | 89.36% |
| S1711 | 6780745 | 6057899 | 89.34% |
| S105 | 6519053 | 5822499 | 89.32% |
| S229 | 6156292 | 5498107 | 89.31% |
| S399 | 6146707 | 5489461 | 89.31% |
| S067 | 6547099 | 5844707 | 89.27% |
| S0410 | 10055252 | 8975271 | 89.26% |
| S056 | 5687419 | 5067327 | 89.10% |
| S1311 | 5407447 | 4818100 | 89.10% |
| S045 | 7958666 | 7089651 | 89.08% |
| S0711 | 8740872 | 7786046 | 89.08% |
| S2810 | 5759881 | 5129932 | 89.06% |
| S029 | 7985198 | 7108176 | 89.02% |
| S335 | 7352310 | 6543950 | 89.01% |
| S2310 | 6670112 | 5936835 | 89.01% |
| S2010 | 5641283 | 5020715 | 89.00% |
| S0611 | 9399814 | 8360736 | 88.95% |
| S3810 | 5613387 | 4987215 | 88.85% |
| S066 | 5177170 | 4599505 | 88.84% |
| S049 | 7725734 | 6862120 | 88.82% |
| S245 | 6989372 | 6204866 | 88.78% |
| S385 | 5321686 | 4724628 | 88.78% |
| S3910 | 5431282 | 4821517 | 88.77% |
| S0610 | 7398240 | 6565427 | 88.74% |
| S057 | 6813910 | 6045888 | 88.73% |
| S015 | 7123103 | 6316634 | 88.68% |
| S115 | 6991600 | 6195712 | 88.62% |
| S2210 | 6052765 | 5354832 | 88.47% |
| S2410 | 5962933 | 5274523 | 88.46% |
| S288 | 7230266 | 6394187 | 88.44% |
| S387 | 6405160 | 5663897 | 88.43% |
| S069 | 6786592 | 6001692 | 88.43% |
| S059 | 7498217 | 6629742 | 88.42% |
| S175 | 6794566 | 6003909 | 88.36% |
| S088 | 6999167 | 6183897 | 88.35% |
| S407 | 7111248 | 6281907 | 88.34% |
| S346 | 7331629 | 6469831 | 88.25% |
| S086 | 6025792 | 5313085 | 88.17% |
| S4010 | 5889301 | 5190958 | 88.14% |
| S017 | 6953015 | 6126269 | 88.11% |
| S1910 | 5973852 | 5262860 | 88.10% |
| S276 | 7767514 | 6837580 | 88.03% |
| S2311 | 7153723 | 6296089 | 88.01% |
| S1710 | 5689222 | 5002703 | 87.93% |
| S2110 | 6756316 | 5938093 | 87.89% |
| S095 | 6052787 | 5313715 | 87.79% |
| S087 | 7277666 | 6385820 | 87.75% |
| S3010 | 6030661 | 5291195 | 87.74% |
| S027 | 7172434 | 6290966 | 87.71% |
| S397 | 8052622 | 7056495 | 87.63% |
| S0511 | 8265444 | 7243333 | 87.63% |
| S3511 | 9213462 | 8057918 | 87.46% |
| S089 | 7421477 | 6478005 | 87.29% |
| S028 | 7479767 | 6522397 | 87.20% |
| S078 | 6722591 | 5860194 | 87.17% |
| S205 | 5834645 | 5078030 | 87.03% |
| S235 | 5900811 | 5132049 | 86.97% |
| S018 | 6955924 | 6048571 | 86.96% |
| S215 | 6494233 | 5636070 | 86.79% |
| S3311 | 9004843 | 7805966 | 86.69% |
| S025 | 7865088 | 6816660 | 86.67% |
| S405 | 5714434 | 4948478 | 86.60% |
| S0210 | 9152381 | 7926132 | 86.60% |
| S386 | 8385475 | 7246030 | 86.41% |
| S0411 | 9616544 | 8303007 | 86.34% |
| S0810 | 8395040 | 7240106 | 86.24% |
| S255 | 6836810 | 5891933 | 86.18% |
| S2510 | 6081083 | 5240624 | 86.18% |
| S286 | 6922128 | 5958527 | 86.08% |
| S409 | 7425042 | 6381951 | 85.95% |
| S135 | 6936580 | 5951772 | 85.80% |
| S0110 | 8745672 | 7499417 | 85.75% |
| S116 | 6221496 | 5329909 | 85.67% |
| S4011 | 6856203 | 5860005 | 85.47% |
| S3911 | 7338541 | 6253603 | 85.22% |
| S216 | 8426880 | 7168789 | 85.07% |
| S098 | 7667020 | 6510544 | 84.92% |
| S0710 | 8118577 | 6887728 | 84.84% |
| S336 | 7895483 | 6679911 | 84.60% |
| S176 | 7483451 | 6318581 | 84.43% |
| S3811 | 7208013 | 6060550 | 84.08% |
| S246 | 7018987 | 5858963 | 83.47% |
| S256 | 6857043 | 5707033 | 83.23% |
| S126 | 6760268 | 5619117 | 83.12% |
| S106 | 7299778 | 6023893 | 82.52% |
| S186 | 6802837 | 5575140 | 81.95% |
| S096 | 5662435 | 4619181 | 81.58% |
| S356 | 7260103 | 5910092 | 81.41% |
| S146 | 6113691 | 4956964 | 81.08% |
| S149 | 5423530 | 4380282 | 80.76% |
| S136 | 6256594 | 5051217 | 80.73% |
| S236 | 6080381 | 4889634 | 80.42% |
| S206 | 7785971 | 6157969 | 79.09% |
| S3411 | 9829261 | 7708476 | 78.42% |
| S148 | 5065702 | 3950065 | 77.98% |
| S145 | 6395950 | 4876589 | 76.24% |
| S147 | 6240715 | 4640116 | 74.35% |
| S1410 | 6091993 | 4459402 | 73.20% |
| NC-EX-16 | 10123 |  |  |
| NC-EX-20 | 45913 |  |  |
| NC-EX-28 | 313355 |  |  |
| NC-JK-16 | 8360 |  |  |
| NC-JK-20 | 51606 |  |  |
| NC-JK-28 | 244730 |  |  |
